# Delirium-Onset of Prodromal Dementia With Lewy Bodies—Putative Brainstem-Related Pathomechanism and Clinical Relevance

**DOI:** 10.3389/fnagi.2022.829098

**Published:** 2022-02-25

**Authors:** Niels Hansen, Charles Timäus, Caroline Bouter, Claudia Lange, Katharina Packroß

**Affiliations:** ^1^Department of Psychiatry and Psychotherapy, University of Goettingen, Göttingen, Germany; ^2^Department of Nuclear Medicine, University Medical Center Göttingen, Göttingen, Germany

**Keywords:** delirium, prodromal dementia with Lewy bodies, locus coeruleus, noradrenaline, alpha-synuclein

## Prodromal Dementia with Lewy Bodies

Dementia with Lewy bodies (DLB) is a prevalent neurodegenerative dementia (Walker et al., 2015). In recent years, early diagnosis of DLB has attracted growing attention as three different onset phenotypes have emerged and crystallized in recent years, such as (1) mild cognitive impairment (MCI)-onset, (2) psychiatric-onset and (3) delirium-onset. Research criteria were proposed by an international consensus group last year (McKeith et al., 2020). These criteria (McKeith et al., 2020) describe the condition necessary to accurately diagnose prodromal DLB with a delirium onset: a delirious episode must precede the occurrence of cognitive decline or dementia. In their condensed guidelines they report that several factors can induce such a delirium episode preceding DLB, similar to the spectrum of factors associated with various types of delirium such as surgery, inflammation, excessive body temperature, alcohol withdrawal, or other types of psychopharmacologic drug withdrawal. They propose (McKeith et al., 2020) that prodromal DLB in particular should be suspected in those cases in which the aforementioned factors typically provoking delirium are not apparent. Furthermore, they suggest special attention be paid whenever a delirium lasts an unusually long time and is not an isolated, single event. They also maintain that it is important that the cognitive impairment be observed after the experience of delirium. However, the authors mention typical caveats (McKeith et al., 2020) to better identify patients with prodromal DLB, namely the low validity of cognitive fluctuations, as this is a characteristic sign of several delirium types and is not specific for prodromal DLB. Furthermore, motor symptoms have to be carefully assessed while keeping in mind the possibility that such symptoms might be the consequence of antipsychotic drug treatment for delirium. Another important point is visual hallucinations, frequently observed in a delirium episode as a consequence of alcohol withdrawal. As the last issue for careful consideration, they point out that rapid eye movement disorder as a key characteristic of DLB is extremely difficult to diagnose in cases of delirium. Finally, the authors conclude that the topic of prodromal DLB with a delirium onset is highly relevant for clinicians as the antipsychotic drug treatment dosage applied in cases of delirium must be carefully reduced if a prodromal DLB is suspected as the delirium's cause. To better diagnose prodromal DLB, biomarkers might be useful (McKeith et al., 2020) such as the use of (123)-I-2-ß-carbomethoxy-3ß-(4-iodophenyl)-N-(3-fluoropropyl) nortropane single photon emission computed tomography (123-CIT SPECT) to diagnose the nigrostriatal degeneration supporting a DLB diagnosis. However, neuroimaging in delirious patients is highly controversial, as severe sedation is often needed to impede the movement artifacts that seriously compromise the quality of such neuroimaging.

## Studies Related to a Delirium Onset of Dementia with Lewy Bodies

The number of large studies reporting delirium as prodromal DLB is limited. FitzGerald et al. (2019) described a large scale study including 194 patients with DLB to define the delirium incidence in their cohort. A year before dementia appeared in these patients, the delirium incidence rate (17.2/100 person years) was much higher than that (3.2/100 person years) observed in 776 Alzheimer dementia (AD) patients. Another investigation years earlier by Vardy et al. reported similar findings (Vardy et al., 2014). They identified a 25% delirium frequency in 85 patients with DLB, whereas a delirium had occurred in just 7% of 95 patients with AD. McKeith et al. (2020), who suggest that a delirium might indicate a prodromal DLB, is further supported by the observation in Vardy's study that more than one delirium episode is reported much more often in conjunction with DLB (23%) than with AD patients (14%). The delirium-onset phenotype is probably more seldom than the psychiatric-onset phenotype with psychotic symptoms. However, no large scale studies exist on this topic. Not a delirium-onset, but delirium was nevertheless observed in 7/21 (33%) of DLB patients during the observation period (Utsumi et al., 2021). Nearly all their patients presented psychotic symptoms as a precursor of DLB (20/21, 95%) as the authors focused on a psychiatric onset of prodromal DLB. One possibility is that prodromal DLB might be followed in the long-run by a delirium episode, as delirium was observed in 33% of prodromal DLB patients suffering a psychotic-onset. In their larger study of 40 screened patients with delirium in a memory outpatient center, 32% had DLB (Hasegawa et al., 2013). However, a delirium episode was as frequent in vascular dementia (34%) as in DLB patients, but delirium was much less frequent in AD patients (15%; Hasegawa et al., 2013). These studies show that a delirium episode reflects a possible DLB manifestation in a subgroup of patients, and that it is much more typical of DLB than of AD. However, other differential diagnoses (like a delirium caused by vascular brain disease) must be systematically ruled out. All in all, the delirium-onset subtype of prodromal DLB seems to occur more often in DLB vs. AD in predementia and dementia stages, although the study evidence is low. A possible explanation why DLB patients in either a predementia or dementia stage are more affected than AD patients might have to do with an impaired autonomic parasympathetic function in DLB caused by damaged autonomic nuclei.

## Putative Pathomechanisms of Brainstem-Related Prodromal DLB with Delirium

**Figure 1:**
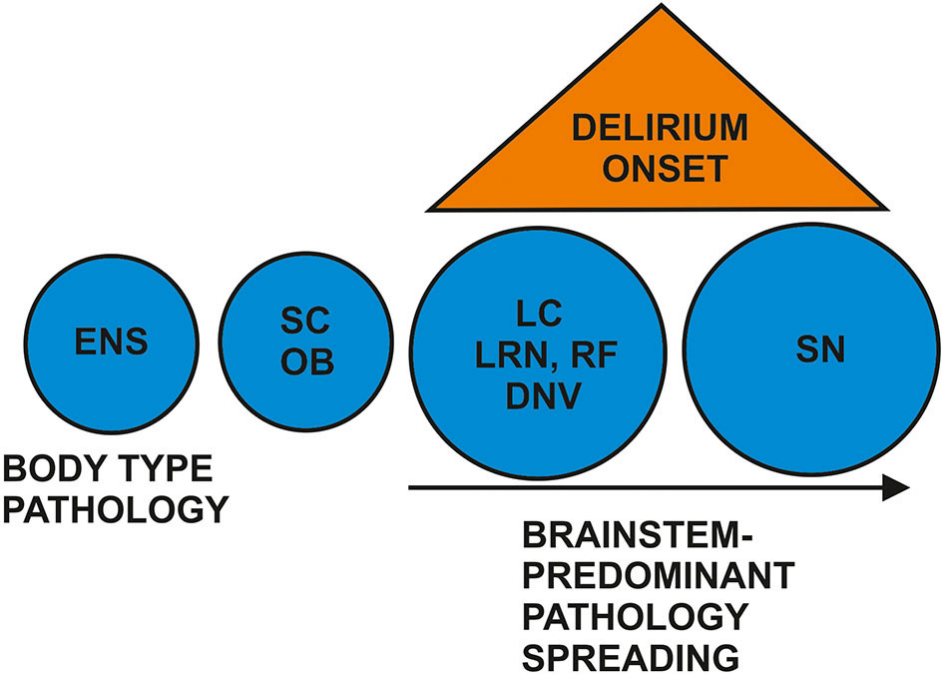
Proposed brainstem predominant pathologies in prodromal DLB's delirium onset. There is controversy about whether neuropathology starts in the enteric nervous system (ENS) as first-body pathology or in the brain such as in the olfactory bulb (OB) or brainstem. However, postulating a brainstem-type of Lewy body pathology in prodromal DLB with delirium, we propose Lewy body pathology and degeneration affecting the spinal cord (SC), dorsal motor nucleus of the vagus nerve (DNV), magnocellular reticular formation (RF), lower raphe nuclei (LRN), and the locus coeruleus (LC). The substantia nigra (SN) is more likely to be affected later than the LC.

Although there are so few studies regarding prodromal DLB with a delirium-onset and DLB patients may start with a delirium episode, the topic has made researchers curious enough to investigate the pathogenic basis of a delirium-onset in DLB patients, although there is no clinical method yet enabling us to differentiate rapidly patients with prodromal DLB and from those with a delirium of another cause. Its neuropathological origin is entirely unknown, and its neuroanatomic pathways seem to be affected differently in prodromal DLB with a delirium-onset than in patients with fully established DLB. A recent study demonstrated Lewy bodies in the substantia nigra (SN) of a minority of patients with delirium—a finding that did not differ in patients without delirium (Davis et al., 2019). The presence of Lewy bodies pathologically reveals the presence of protein alpha synuclein (Burré et al., 2018). The absence of Lewy bodies in the SN could be attributable to initial Lewy body deposited in another brainstem location prior to SN deposition of Lewy bodies, i.e., in the locus coeruleus (LC), as reported recently in a recent review (Hansen, 2021). However, the Lewy body pathology in the LC and SN is one of the potentially diverse patterns of the various anatomical pathways affected early in Lewy body disease (Spotorno et al., 2020; Borghammer et al., 2021) such as an affected olfactory bulb, amygdala with possible olfactory bulb involvement, brainstem with possible amygdala or olfactory bulb contribution, the limbic system (possible olfactory bulb, amygdala and brainstem contribution) and the neocortical pattern with potential involvement of all other aforementioned predilection sites (Attems et al., 2021). In other words, not all patients with Lewy body pathology adopt the same pathological spreading pattern of alpha-synucleinopathies. Indeed, a pattern is often observed with affected structures such as the amygdala, entorhinal cortex and SN, but other patterns such as the involvement of lower brainstem nuclei and autonomous nuclei are another potential pattern in Lewy body pathology (Borghammer et al., 2021) termed as a predominantly brainstem pattern (Attems et al., 2021), so that the LC's involvement in delirium could be one possible, but not the sole conceivable pattern. Furthermore, a key question distinguishing different Lewy body-pathology patterns and their spread is the existence of an Alzheimer co-pathology, as one investigation (Spotorno et al., 2020) demonstrated that those LBD patients presenting such an Alzheimer co-pathology exhibit cortical thinning more often in the temporal lobe. Thus, there is substantial debate about which time relationship and in what type of pattern the pathological Lewy body pathology spreads, and how this relates to the prodromal DLB phenotype. For instance, a recent study (Chen et al., 2021) demonstrated that the SN displayed an early pathology also in patients with MCI and Lewy body pathology vs. controls in 3 T MRI analyzed via quantitative susceptibility mapping. Another working group (Saari et al., 2021) showed that those patients with more depressive symptoms and LBD had more sparsely distributed neurons than those with no depressive symptoms. Thus, further research is needed into how certain clinical features are associated with which type of Lewy body pathology and its spread. The absence of Lewy bodies in the SN could thus implicate Lewy bodies in lower brainstem nuclei such as the LC. Lewy body pathology might start in the enteric nervous system (Borghammer et al., 2021), but it is also found in the brain, i.e., the olfactory bulb, brainstem, amygdala, limbic or neocortical pattern, as evident in recent neuropathological consensus guidelines termed LPC criteria (LP = Lewy body pathology relating to Lewy bodies and Lewy neurites, C = consensus criteria; Attems et al., 2021). Therefore, Lewy bodies deposited in the LC is one route of Lewy body pathology distribution, but one not applying to all cases of prodromal DLB. More clinicopathological investigations should be done to clarify this issue. However, if the LC is affected by LC pathology (brainstem-predominant type of Lewy body pathology), the deposition of Lewy bodies in structures such as the LC might have functional consequences for the release of noradrenaline, as (1) the LC is the main source of noradrenaline in the brain and (2), noradrenergic neurons degenerate in the LC with a compensatorily altered release pattern of noradrenaline in the brain. Brain functions involved in the noradrenergic processing of information are thus altered by Lewy-body pathology, like the attention and executive functions typically affected in DLB. It is potentially worthwhile to assess the olfactory function in delirium patients suspected of having DLB via sniffing sticks, as there is recent evidence (Kon et al., 2020) that Lewy body pathology might affect the olfactory bulb even before it affects the LC. Alpha synuclein might accumulate via Lewy bodies in specific neuroanatomical pathways as aforementioned, such as the first the olfactory bulb, amygdala, or brainstem-predominant pathway including the LC and substantia nigra, then the limbic pathway, and finally the diffuse neocortical pathway; this is the neuropathological hallmark of DLB and its time course of disease progression (Lashuel et al., 2013; Burré et al., 2018; Kon et al., 2020). However, as mentioned above, there may be other specific anatomic predilection patterns controlling how alpha-synucleinopathy spreads within the brain or secondarily in the brain after early manifestation in the enteric nervous system. In patients suffering a postoperative delirium (POD) following hip fracture surgery or a gastrectomy, two studies (Sunwoo et al., 2013; Yuan et al., 2020) demonstrated that exosome alpha synuclein in blood plasma (Yuan et al., 2020) and alpha-synuclein immunoreactivity in myenteric plexus (Sunwoo et al., 2013) were higher in patients with POD than in those without POD. Their evidence indicates that early alpha-synuclein production or deposition might play a decisive role in generating delirium in surgery patients who already have alpha-synucleinopathy, or that delirium triggers increased accumulation of alpha-synuclein in blood plasma or the myenteric plexus in those patients. Alpha-synucleinopathy in a brainstem location as one possible route for alpha-synucleinopathy's spreading pattern might peak or exceed a specific limit beyond which it triggers delirium states via systemic inflammation such as sepsis, inflammatory conditions, or postoperative disorders; thus a delirium symptomatology would appear in patients that could facilitate a prodromal DLB diagnosis. Lewy body pathology's effects in structures such as the LC or other brainstem structures (Seidel et al., 2015) like the reticular formation or dorsal vagus nerve (DVN) (Del Tredici and Braak, 2013) may explain why attention, cognitive, arousal, and vegetative functions as well as consciousness are impaired in patients with a delirium-onset and possible prodromal DLB. We propose that the brain pathology in DLB's delirium onset might resemble one predominant brainstem-affection subtype with its initial pathology in structures such as the olfactory bulb (OB), peripheral nervous system (PNS) like myenteric plexus, and DVN, as well as later involvement of the LC. As the time relationship as to when structures are affected by Lewy body pathology it is unknown, this question should be kept open and further studies done to shed light on this issue. We do not yet know whether patients with a psychiatric-onset, delirium-onset or MCI-onset of prodromal DLB differ in their brainstem pathology—a subject that deserves further investigation in animal and human studies, including postmortem brain histopathology. A recent study showed that patients with MCI and hints for DLB also presented volume reduction in the nucleus basalis Meynert that did not differ from other MCI patients such as those with Alzheimer's pathology (Schumacher et al., 2021) indicating that prodromal DLB with the MCI subtype might entail a degenerated nucleus basalis Meynert. That would make possible that the nucleus basalis Meynert is affected in prodromal DLB with an MCI onset. Recent studies have not been specific in declaring the localization of Lewy body pathology and how it correlates with clinical features. It does not always follow the same bottom-up progression from brainstem to cortex, as there may also be a multifocal distributional pattern of Lewy body pathology (Uchihara and Giasson, 2016). A recent study differentiated between a body-first subtype vs. a brain-first subtype of Lewy body pathology, both including Lewy body pathology within the LC, of different extents of severity and time points coinciding with the Lewy pathology (Borghammer et al., 2021). LC degeneration occurs in the prodromal phase of DLB in the body-first type, but LC degeneration in the brain-first type is detected after a DLB diagnosis (Borghammer et al., 2021). Both anatomical predilection subtypes, either the caudo-rostral or amygdala-centered pattern, might include LC degeneration. However, degeneration of autonomic nuclei, sympathetic and parasympathetic denervation occurs mainly in the body-first subtype affecting the brainstem also including the LC. The delirium phenotype shares several features related to a dysregulated vegetative nervous system, making it likely that the brainstem-predominant pattern might be detected in prodromal DLB with a delirium-onset that we propose (see Figure 1 for schematic delineation of LC involvement in delirium-onset prodromal DLB). More investigation should be done to assess the relevance and frequency of these patterns of Lewy body pathology in prodromal DLB, and exactly how and when they spread.

## Final Remarks and Conclusion

We are planning a study to assess the risk factors for delirium (Find delirium risk factors = FINDERI, Trial registration: German Clinical Trials Register: DRKS00025095; Sadlonova et al., 2021) and the frequency of the delirium-onset subtype of prodromal DLB in conjunction with POD in a cohort of cardiac-surgery patients. Furthermore, we will determine cell destruction markers to measure neural brain damage, and seek potentially useful biomarkers. We maintain that prodromal DLB's delirium onset is a neglected entity, and that further research is essential to identify such patients early to improve their therapy. Delirium is a frequent phenomenon in intermediate care units, and often treated with antipsychotic drugs. However, the use of antipsychotic drugs might trigger adverse reactions in DLB patients. It is therefore very clinically relevant to further develop biomarkers to identify these patients at an early stage so as to prevent the serious adverse sequelae that drug therapy can cause.

## Author Contributions

NH wrote and conceptualized the manuscript. CT, CB, CL, and KP revised the manuscript for important intellectual content. All authors contributed to the article and approved the submitted version.

## Funding

Funding was received from the Open access fund of the University of Göttingen.

## Conflict of Interest

The authors declare that the research was conducted in the absence of any commercial or financial relationships that could be construed as a potential conflict of interest.

## Publisher's Note

All claims expressed in this article are solely those of the authors and do not necessarily represent those of their affiliated organizations, or those of the publisher, the editors and the reviewers. Any product that may be evaluated in this article, or claim that may be made by its manufacturer, is not guaranteed or endorsed by the publisher.
